# Perceptions about brain health among the United Arab Emirates population using the global brain survey: a cross-sectional study

**DOI:** 10.3389/fpubh.2025.1518938

**Published:** 2025-03-11

**Authors:** Iffat Elbarazi, Aminu S. Abdullahi, Karim Abdel Aziz, Emmanuel Stip, Ismail Elkonaisi, Fayeza Fahim, Maryum Asghar, Isabelle Budin-Ljøsne, Javaid Nauman

**Affiliations:** ^1^Institute of Public Health, College of Medicine and Health Sciences, United Arab Emirates University, Al-Ain, United Arab Emirates; ^2^Department of Psychiatry, College of Medicine and Health Sciences, United Arab Emirates University, Al-Ain, United Arab Emirates; ^3^Department of Psychiatry, University of Montreal, Montreal, QC, Canada; ^4^Department of Food Safety, Norwegian Institute of Public Health, Oslo, Norway; ^5^Department of Circulation and Medical Imaging, Faculty of Medicine and Health Sciences, Norwegian University of Science and Technology, Trondheim, Norway; ^6^Healthy Living for Pandemic Event Protection (HL-PIVOT) Network, Chicago, IL, United States

**Keywords:** brain health, mental health, perceptions, health knowledge, United Arab Emirates (UAE), Global Brain Health Survey

## Abstract

**Introduction:**

Interest in brain health and general well-being research has increased due to advances in neurosciences, and aging population’s need for preventive health measures. However, there is limited research on perceptions and attitudes toward brain health in the United Arab Emirates (UAE), a country with a unique demographic and cultural context. We aimed to assess self-reported practices, beliefs and attitudes toward brain health within the UAE population, identifying key factors influencing these views, and contributing to the global understanding of brain health in non-Western, high-income settings.

**Methods:**

We conducted a cross-sectional study using the UAE-adapted Global Brain Health Survey, originally developed by the Lifebrain Consortium in Europe. The survey was distributed in both English and Arabic language via social media and the snowball technique. Data analysis included descriptive statistics and results of multivariable binary logistic regression.

**Results:**

A total of 931 responses were recorded and analyzed. Overall, participants demonstrated a moderate understanding of brain health. Key factors that participants believed to influence brain health were physical health, sleep habits, substance use, and social environment. Older participants were more likely to engage in healthy lifestyle choices, while younger participants prioritized different activities. We also observed gender differences, with women less likely to engage in activities such as taking nutritional supplements and practicing relaxation techniques. Higher education and healthcare experience were linked to more informed perceptions of brain health.

**Discussion:**

Our findings provide valuable insights into how cultural, social, and demographic factors influence the practices and beliefs toward brain health in the UAE. By adapting the Global Brain Health Survey for a Middle Eastern context, we demonstrate its cross-cultural applicability and contribute to the global discourse on brain health. Our results may inform future public health policies and interventions, highlighting the importance of tailored culturally sensitive strategies to promote brain health across different demographic groups, particularly in multicultural and rapidly aging societies.

## Highlights

- *Main findings* - Physical health, sleep habits, substance use, and social environment are key factors influencing brain health perceptions in the UAE, with notable differences across age, gender, education, and healthcare experience.
- *Added knowledge* - Our study is a first comprehensive assessment of self-reported practices and beliefs about factors influencing brain health in the UAE, filling a gap in regional studies by highlighting demographic disparities, and expanding the global understanding of brain health perceptions.
- *Public health impact for policy and action* - The study findings can guide the development of culturally and demographically tailored brain health initiatives and public health policies, which can be adapted globally to enhance brain health awareness and promote preventive measures in diverse populations.

## Background

Brain health is a relatively a new concept, comprising both mental and cognitive health, wherein a healthy brain involves not only the absence of brain disease but also mental wellbeing and normal functioning of the brain (1). Interest in research on brain health and general wellbeing has been on the increase possibly due to the increased aging population worldwide, development in the field of neuroscience and technology, and the growing awareness about physical and mental health (2). Conceptualizing brain health has been challenging, due to the its complex nature where most definitions have described brain health as normal functioning of brain from a physical perspective being an organ (3, 4). Chen et al. defined brain health as: “a multidimensional, dynamic state, occurring along the life course, and incorporating cognitive, emotional, and motor domains underpinned by physiological processes,” while also stating that setting a concrete definition would create boundaries and lack inclusivity for a matter as complex as brain health (2). More contemporary definitions have taken into consideration the mental and the cognitive components. The Center for Disease Control and Prevention (CDC) defined brain health as “an ability to perform all the mental processes of cognition, including the ability to learn and judge, use language and remember” (4). This can be described as the preservation of the integrity of the brain as an organ, its mental and cognitive function, with the absence of a neurological disorders (3). The operational definition of brain health in the present study is similar to as previously stated (1) being a comprehensive concept to describe both neurological and mental health disorders including “health conditions related to function, diseases, injuries and disorders of the brain and other parts of the nervous system.”

Brain related disorders are also expected to increase due to the increased life expectancy (5). The results of Global Burden of Disease Study in 2016 confirmed that neurological disorders were the leading cause of disability worldwide, and accounted for about 276 million disability adjusted life years (6). The number of dementia cases globally are also forecasted to increase from 57.4 million in 2019 to 152.8 million by 2050, with a higher female to male ratio (7). Previous studies also highlighted the importance of early interventions and lifestyle changes, especially with certain brain conditions such as Alzheimer disease, dementia and others, showing that about 40% of dementia cases might be prevented by lifestyle choices (5, 8).

A gap in research has been noted, with respect to perception of population around the world about brain health and the steps that are needed to be taken to maintain healthy brain (9, 10). Surveys about brain health awareness conducted previously in different parts of the world at national levels, have suggested the limited understanding by the public about conditions such as dementia, schizophrenia, and factors contributing to it, and the reluctance of general population to develop habits that would promote brain health (11, 12).

The Lifebrain- a European consortium- specializing in research based on brain health by analyzing data from brain imaging cohorts across eight European countries conducted a study called Global Brain Health Survey (GBHS) (9) in the period of June 2019–August 2020 in 81 countries with the aim of exploring people’s perception of brain health in a quest to find ways to improve brain health, and to explore the support they need to make the necessary lifestyle changes to lead a healthier brain. The study results endorsed the need for public health interventions through evidence-based research in brain health, the need for more professional support, and the introduction of exercise and physical activity in schools and work places and subsidies on healthy food to promote a healthier lifestyle (1, 9).

Studies conducted my Maalouf et al., in 2019 noted that although the Arab world contributes to 5.4% of global disabilities due to neurological disorders, it contributes only 1% of the global output of peer reviewed publications (13). Few of the brain health research challenges identified in the Arab world were stigma associated around mental health, lack of knowledge of healthy lifestyle and cognitive function, and brain health impact due to war and armed conflict (13). Al-Krenawi et al., conducted a systematic review covering mental health in Arab world and found that patients with mental health in Arab countries do not express their mental health issues, and do not visit health care services due to stigma with more manifestation of mental health issues in a form of psychosomatics (14).

The United Arab Emirates (UAE) is a country with diverse population with the national citizens (Emiratis) constitute only 11% of the total population while the rest is distributed between 200 nationalities, majorities coming from South Asia (15). The literature surrounding perception of brain health by the general population in the Arab world in general, and in the UAE in particular, is scarce. Despite the increase in the healthcare development in the last three decades, mental health or brain health programs are still scarce and highly needed (16). Therefore, the aim of the present study was to contribute to the global understanding of brain health in the UAE – a non-Western, high-income country with a unique demographic and cultural context. The focused research questions in our study were related to practices, beliefs and attitudes toward brain health, and how these practices and beliefs are influenced by the demographic and lifestyle factors. We hypothesized that higher education and professional experiences in healthcare industry will be associated with more informed perceptions of brain health, and women and older adults will demonstrate varying perceptions and attitudes toward brain health compared with men and younger groups.

Our study is grounded in the socio-ecological model, which emphasizes the interplay between individual, social, and environmental factors in shaping health behaviors and perceptions. This framework is particularly relevant for understanding brain health attitudes in the UAE’s multicultural context, where cultural norms, social structures, and environmental influences significantly impact health-related decisions.

## Methods and materials

This is a cross-sectional study that was conducted within the UAE, building upon the updated and adapted GBHS developed by the Lifebrain Consortium (1, 9). We adopted a convenience sampling strategy to recruit participants, distributing the survey both online and in person. The Arabic and English questionnaires were hosted on SurveyMonkey and shared via widely used social media platforms, including Facebook, Twitter, Instagram, and LinkedIn, as well as WhatsApp groups. These platforms targeted general population, university networks, professional communities, and local forums to ensure broad participation. Additionally, printed flyers with QR codes linking to the survey were distributed in universities, shopping malls, coffee shops, and workplaces. The research team also approached their personal and professional networks to disseminate the survey through email invitations and direct messaging. We employed this approach to maximize accessibility and participation, capturing a diverse range of respondents across the UAE. Data collection took place from September 2021 to January 2022. The study received approval from the original creators, the Lifebrain Consortium, and was ethically approved by the UAE University Social Science Ethics Committee (ERS_2020_7231).

### The questionnaire

Using the GBHS, we investigated the participants perceptions and attitudes toward brain health, their intentional efforts to enhance or sustain brain health, their motivation to undergo brain health assessments, their willingness (or unwillingness) to participate in brain-boosting activities, and the type of support they would require making lifestyle changes. Additionally, we probed respondents to identify the most effective public health measures for promoting brain health.

Given the multifaceted social, religious, and cultural landscape of the UAE, we expanded the GBHS questionnaire to encompass questions pertaining to religious and cultural practices. Following feedback from the Lifebrain Consortium on these supplementary questions, we translated the questionnaire into Arabic language. This involved a collaborative translation process conducted by two native Arabic speakers working in tandem. Subsequently, we piloted the Arabic version of the questionnaire with a group of 25 participants, making necessary refinements based on their feedback. To ensure accuracy and content validity, the Arabic version of the questionnaire underwent thorough review and piloting by native Arabic speakers. The adapted questionnaire started with an informative section outlining the survey’s purpose and primary objectives. It included questions related to demographic information, as well as participants’ knowledge, beliefs, and attitudes concerning brain health. The survey also explored lifestyle practices that are either followed or believed to contribute to enhanced brain health.

We added additional questions in the survey to account for the participants’ broad social, religious, and cultural diversity. These supplementary questions and relevant response options were: (1) “Imagine undergoing a brain health test that indicates a risk of developing a brain disease. Would seeking assistance from a religious person or healer be your most probable reaction?” Response options range from “definitely yes” to “definitely no.” (2) “Your doctor suggests that changing your lifestyle can lower your risk of developing a brain disease. How likely are you to increase your involvement in cultural/religious activities?” Choices consist of: “very likely,” “somewhat likely,” “I already do that,” “somewhat unlikely,” “very unlikely,” and “not applicable.” (3) “Suppose you decide to modify your lifestyle to enhance or maintain brain health. What form of assistance would you consider?” One of the options is “support from a religious organization. (4) “What actions have you taken to promote brain health during the COVID-19 pandemic?” Among the options, “religious activities (pray, script reading, go to worship places)” is included, and respondents were asked to assess the frequency with responses including “frequently,” “occasionally,” “rarely,” and “never.”

### Ethics and consent

The study was approved by the United Arab Emirates Social Science Ethics Committee. Approval number (ERS_2020_7231). The participation in the study was on volunteer basis, and to encourage participation, an Instagram competition offered the chance to win gift vouchers. Upon participation, the winners of these vouchers were selected through a raffle process. All individuals provided written informed consent to participate in the study, and to use the collected data for research purposes.

### Data analysis

Categorical variables were summarized using frequencies and percentages. Age categories were collapsed into three: 18–40 years, 41–60 years, and above 60 years. Nationality of the participants was presented as a dichotomous variable with “Emiratis” and “others.” Highest education level was presented in two categories for easy interpretation. The first category is “below university” which includes all levels of education below university level, and the second is “above university” including all university-level education. Responses to self-rated mental health and self-rated cognitive health were merged into two categories – “average and above” (including “excellent,” “above average,” and “average”) and “below average” (including “below average” and “very poor”).

Multivariable binary logistic regression models were used to identify demographic factors associated with various activities performed by the participants purposefully for brain health, factors perceived to strongly influence brain health, and beliefs regarding the stage of life important to take special care of the brain. Independent factors explored were age, gender, education, and healthcare experience. For factors perceived to influence brain health, each factor has five possible responses originally including “very strong influence,” “strong influence,” “moderate influence,” “weak influence,” and “no influence.” These were collapsed into two categories thus “very strong influence,” and “strong influence” were categorized as having a positive belief in the strong influence the particular factor has on brain health. Regarding stages in life in which it is important to take special care of the brain, the responses “very important” and “important” were categorized as having the opinion that the particular stage of life has high importance. For activities performed purposefully for brain health, “frequently” and “occasionally” responses were considered to indicate a participant performs those activities purposefully for their brain health. The multivariable adjustment was made for all the independent factors in the models to address potential confounding. Adjusted odds ratios and the corresponding 95% confidence intervals were reported. In our analyses, the overall missing data was minimal (<7%), and we adopted pairwise deletion approach (available-case analysis) to address missing data. We included only those participants with available responses at each stage of the analysis to avoid unnecessary exclusion and retain the power of the study as much as possible. The data was analyzed using the IBM Statistical SPSS software version 28 and R software version 4.1.2. We adhered to the STROBE cross-sectional reporting guidelines for presentation of the results.

## Results

### Participants’ characteristics

In total, 931 people participated in the survey with the majority (73%) between the age of 18 and 40 years (Table 1). About two-thirds (65%) were females. Of the respondents, 31% were Emiratis. Most of the participants reside in Abu Dhabi Emirate (73%). University-level education was reported by 62% of the participants. Moreover, 42% were either educated and/or had experience in healthcare. More than half (57%) were single or in a stable relationship. Only a handful of the participants (9%) participated in brain health research before. Most rated their cognitive health (99%) and mental health (90%) as average or higher. 17% suffer from one or the other chronic disease. Only 22% reported having experience looking after a family member with brain disease.

**Table 1:** Characteristics of the participants (*N* = 931).

Characteristic	Frequency (%)
Age	
18–40	675 (73%)
41–60	214 (23%)
>60	42 (4.5%)
Gender	
Male	326 (35%)
Female	595 (65%)
Nationality	
Emirati	109 (31%)
Others	245 (69%)
Emirate	
Abu Dhabi	637 (73%)
Dubai	108 (12%)
Sharjah	43 (4.9%)
Others	90 (10%)
Highest education	
Below university	332 (38%)
University	540 (62%)
Marital status	
Single/stable relationship	489 (57%)
Married	357 (41%)
Divorced/separated/widowed	19 (2%)
Healthcare educated or worker	
Yes	363 (42%)
No	509 (58%)
Ever participated in brain health research	
Yes	82 (9%)
No	849 (91%)
Self-rated cognitive health	
Average and above	865 (99%)
Below average	7 (1%)
Self-rated mental health	
Average and above	781 (90%)
Below average	91 (10%)
Have chronic illness	
Yes	147 (17%)
No	725 (83%)
Experience looking after a family member with brain disease	
Yes	72 (22%)
No	259 (78%)

### Activities for brain health

**Figure 1:**
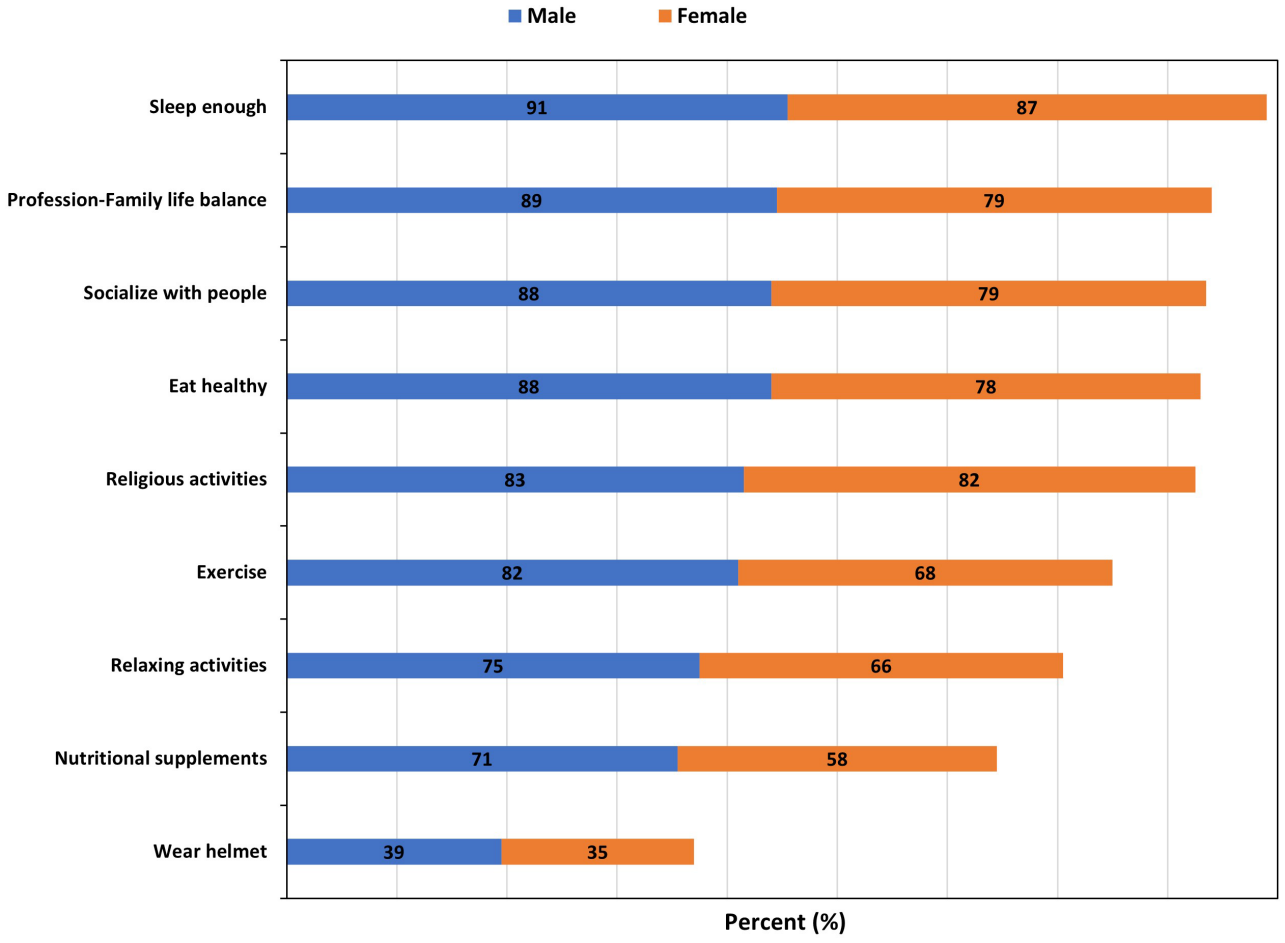
Activities done purposefully for brain health. Number presented represent the percentages (%).

Having enough sleep was the most common activity performed purposefully for brain health by both males (91%) and females (87%) (Figure 1). This is followed by profession-family life balance (89%) and religious activities (82%) among males and females, respectively. Most of the males (83%) also reported religious activities as an activity they perform for brain health. Wearing a helmet was the least common activity performed for brain health in both males (39%) and females (35%).

Participants who believed certain activities – including taking good diet, having good sleeping habits, and engaging in religious activities/practices – to influence brain health were significantly more likely to practice those activities purposefully for their brain health compared to those who did not attach such importance to those activities as influencers of brain health (Table 2). Compared with the younger participants, those above 40 years were significantly more likely to, purposefully for brain health, eat healthy (OR = 2.58, 95% CI = 1.55–4.49), have enough sleep (OR = 2.00, 95% CI = 1.14–3.72), practice relaxing activities (OR = 1.49, 95% CI = 1.04–2.16), maintain profession-family life balance (OR = 2.3, 95% CI = 1.38–4.01), and take nutritional supplements (OR = 2.49, 95% CI = 1.73–3.65); and were less likely to wear helmet (OR = 0.64, 95% CI = 0.45–0.90) (Table 3). Women were less likely than men to, for the purpose of brain health, eat healthy (OR = 0.61, 95% CI = 0.40–0.92), maintain profession-family life balance (OR = 0.56, 95% CI = 0.35–0.86), take nutritional supplements (OR = 0.69, 95% CI = 0.50–0.95), and practice relaxing activities (OR = 0.57, 95% CI = 0.37–0.87) (Table 3). A detailed summary of responses with regard to activities purposefully carried out by the participants for brain health is provided in Supplementary Table 1.

**Table 2:** Activities believed by the participants to influence brain health and practice of such activities by the participants purposefully for brain health.

Believed to influence brain health	Practiced purposefully for brain health		*p*-value
	Yes	No	
Diet influences brain health			0.005
Yes	620 (83%)	130 (17%)	
No	124 (73%)	45 (27%)	
Good sleeping habits			0.003
Yes	745 (89%)	89 (11%)	
No	76 (79%)	20 (21%)	
Religious activities or practices			<0.001
Yes	626 (89%)	76 (11%)	
No	140 (61%)	89 (39%)	
Socializing with people			0.900
Yes	656 (82%)	140 (18%)	
No	109 (82%)	24 (18%)	

**Table 3:** Association between demographics and activities done purposefully for brain health.

Factors	Eat healthy		Exercise		Sleep enough		Relaxing activities	
	OR	95% CI	OR	95% CI	OR	95% CI	OR	95% CI
Age								
18–40	1.00		1.00		1.00		1.00	
>40	**2.58**	**1.55, 4.49**	1.17	0.80, 1.74	**2.00**	**1.14, 3.72**	**1.49**	**1.04, 2.16**
Gender								
Men	1.00		1.00		1.00		1.00	
Women	**0.61**	**0.40, 0.92**	0.50	0.34, 0.72	0.91	0.56, 1.46	0.83	0.59, 1.15
Education								
Lower	1.00		1.00		1.00		1.00	
Higher	1.29	0.90, 1.85	1.36	0.99, 1.86	1.19	0.77, 1.82	0.92	0.67, 1.24
Healthcare experience								
No	1.00		1.00		1.00		1.00	
Yes	1.07	0.75, 1.54	1.26	0.92, 1.73	0.77	0.51, 1.18	0.93	0.69, 1.25

Factors	Profession-family life balance		Wear helmet		Nutritional supplements		Socialize	
	OR	95% CI	OR	95% CI	OR	95% CI	OR	95% CI
Age								
18–40	1.00		1.00		1.00		1.00	
>40	**2.30**	**1.38, 4.01**	**0.64**	**0.45, 0.90**	**2.49**	**1.73, 3.65**	1.31	0.84, 2.08
Gender								
Men	1.00		1.00		1.00		1.00	
Women	**0.56**	**0.35, 0.86**	0.81	0.59, 1.11	**0.69**	**0.50, 0.95**	**0.57**	**0.37, 0.87**
Education								
Lower	1.00		1.00		1.00		1.00	
Higher	1.23	0.85, 1.78	1.20	0.90, 1.62	0.93	0.69, 1.25	0.94	0.65, 1.36
Healthcare experience								
No	1.00		1.00		1.00		1.00	
Yes	1.00	0.69, 1.45	1.27	0.96, 1.68	1.21	0.91, 1.63	1.00	0.70, 1.44

OR, odds ratios; CI, confidence intervals.

OR are adjusted for age, gender, education and healthcare experience.

Bold values are significant at *P* < 0.05.

### Factors perceived to influence brain health

**Figure 2:**
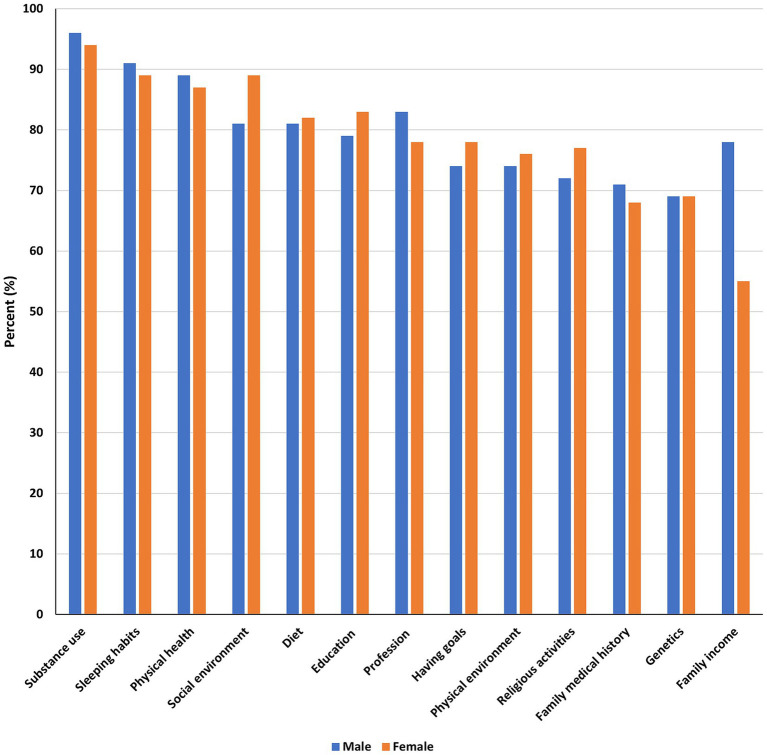
Factors perceived to strongly influence brain health by gender.

Factors perceived to influence brain health strongly are summarized by gender in Figure 2. Substance abuse was the most common factor believed to influence brain health among both males (96%) and females (94%), followed by sleeping habits – males (91%), and females (89%). The least perceived brain health influencing factors for males (69%) and females (55%) were genetics and family income, respectively.

Being 40 years or older was significantly associated with the belief that sleeping habits (OR = 1.94, 95% CI = 1.07–3.77), physical environment (OR = 1.55, 95% CI = 1.05–2.32), family history (OR = 1.47, 95% CI = 1.03–2.12), and family income (OR = 1.72, 95% CI = 1.19–2.50) strongly influence brain health compared to those below the age of 40 (Table 4). On the other hand, these older participants were less likely to believe in the influence of education (OR = 0.60, 95% CI = 0.40–0.88), and having goals (OR = 0.61, 95% CI = 0.43–0.88) for brain health. Compared to men, women were more likely to believe in social environment influence on brain health (OR = 1.64, 95% CI = 1.08–2.48), and less likely to believe in family income influence on brain health (OR = 0.40, 95% CI = 0.28–0.56). Having a higher education level was significantly associated with the perception that physical health (OR = 1.75, 95% CI = 1.16–2.63), diet (OR = 1.69, 95% CI = 1.05–2.42), social environment (OR = 2.18, 95% CI = 1.48–3.24), having goals (OR = 1.49, 95% CI = 1.08–2.06), and physical environment (OR = 1.59, 95% CI = 1.16–2.19) strongly influence brain health. Participants with healthcare experience believed more on the influence of sleeping habits (OR = 1.62, 95% CI = 1.13–2.39), diet (OR = 1.64, 95% CI = 1.13–2.39), and family income (OR = 1.71, 95% CI = 1.27–2.31) than those without healthcare experience. A detailed summary of responses to the question on factors perceived to strongly influence brain health is provided in Supplementary Table 2.

**Table 4:** Association between demographics and factors believed to influence brain health strongly.

Factors	Substance use		Sleeping habits		Physical health		Social environment	
	OR	95% CI	OR	95% CI	OR	95% CI	OR	95% CI
Age								
18–40	1.00		1.00		1.00		1.00	
>40	2.19	0.94, 6.01	**1.94**	**1.07, 3.77**	1.37	0.82, 2.36	0.84	0.54, 1.33
Gender								
Men	1.00		1.00		1.00		1.00	
Women	0.71	0.33, 1.43	0.97	0.58, 1.61	0.83	0.52, 1.30	**1.64**	**1.08, 2.48**
Education								
Lower	1.00		1.00		1.00		1.00	
Higher	1.05	0.56, 1.95	1.15	0.72, 1.81	**1.75**	**1.16, 2.63**	**2.18**	**1.48, 3.24**
Healthcare experience								
No	1.00		1.00		1.00		1.00	
Yes	1.14	0.62, 2.15	**1.62**	**1.01, 2.64**	1.45	0.96, 2.23	1.00	0.68, 1.49

Factors	Diet		Education		Profession		Having goals	
	OR	95% CI	OR	95% CI	OR	95% CI	OR	95% CI
Age								
18–40	1.00		1.00		1.00		1.00	
>40	1.23	0.80, 1.94	**0.60**	**0.40, 0.88**	1.21	0.80, 1.85	**0.61**	**0.43, 0.88**
Gender								
Men	1.00		1.00		1.00		1.00	
Women	1.14	0.77, 1.68	1.19	0.81, 1.72	0.77	0.52, 1.12	0.99	0.70, 1.40
Education								
Lower	1.00		1.00		1.00		1.00	
Higher	**1.69**	**1.18, 2.42**	1.12	0.78, 1.60	1.21	0.86, 1.70	**1.49**	**1.08, 2.06**
Healthcare experience								
No	1.00		1.00		1.00		1.00	
Yes	**1.64**	**1.13, 2.39**	0.71	0.50, 1.00	0.86	0.61, 1.20	0.82	0.60, 1.13

Factors	Physical environment		Family history		Genetics		Family income	
	AOR	95% CI	AOR	95% CI	AOR	95% CI	AOR	95% CI
Age								
18–40	1.00		1.00		1.00		1.00	
>40	**1.55**	**1.05, 2.32**	**1.47**	**1.03, 2.12**	1.39	0.97, 1.99	**1.72**	**1.19, 2.50**
Gender								
Men	1.00		1.00		1.00		1.00	
Women	1.26	0.89, 1.79	0.95	0.68, 1.32	1.13	0.82, 1.56	**0.40**	**0.28, 0.56**
Education								
Lower	1.00		1.00		1.00		1.00	
Higher	**1.59**	**1.16, 2.19**	0.97	0.72, 1.31	1.01	0.75, 1.36	0.82	0.61, 1.11
Healthcare experience								
No	1.00		1.00		1.00		1.00	
Yes	1.14	0.83, 1.58	1.17	0.87, 1.57	1.10	0.82, 1.48	**1.71**	**1.27, 2.31**

OR, odds ratios; CI, confidence intervals.

OR are adjusted for age, gender, education and healthcare experience.

Bold values are significant at *P* < 0.05.

**Figure 3:**
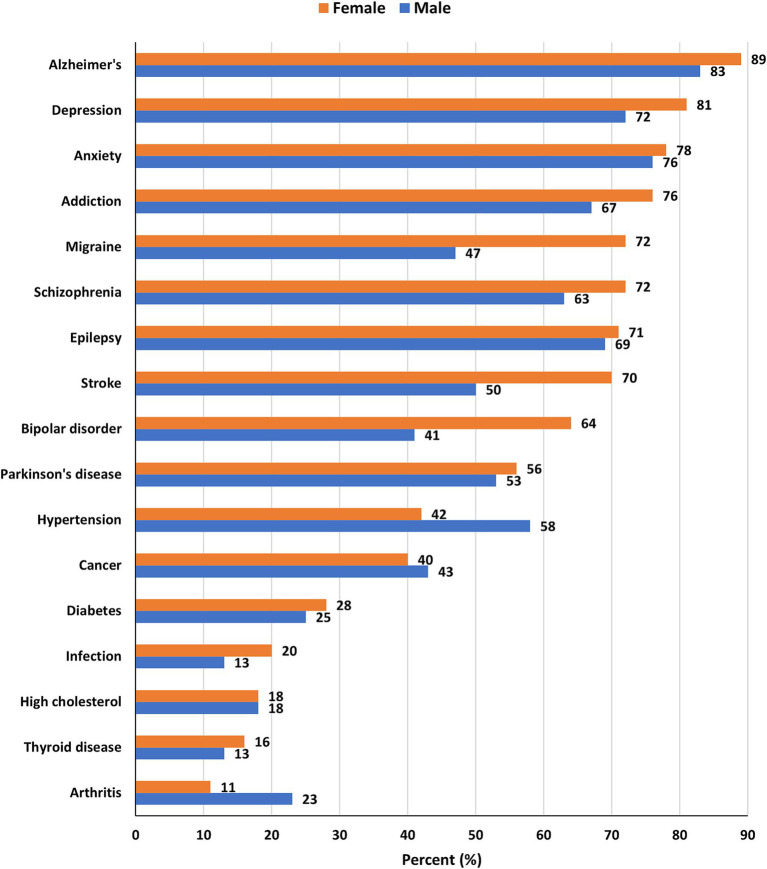
Diseases believed to be associated with brain health by gender.

When asked about diseases believed to be associated with brain health, most male and female participants mentioned mental health disorders including Alzheimer’s (89% females, 83% males), depression (81% females, 72% males), and anxiety (78% females, 76% males). A full summary of the diseases and responses by gender is illustrated in Figure 3.

### Opinions on the stage in life to take special care of the brain

**Figure 4:**
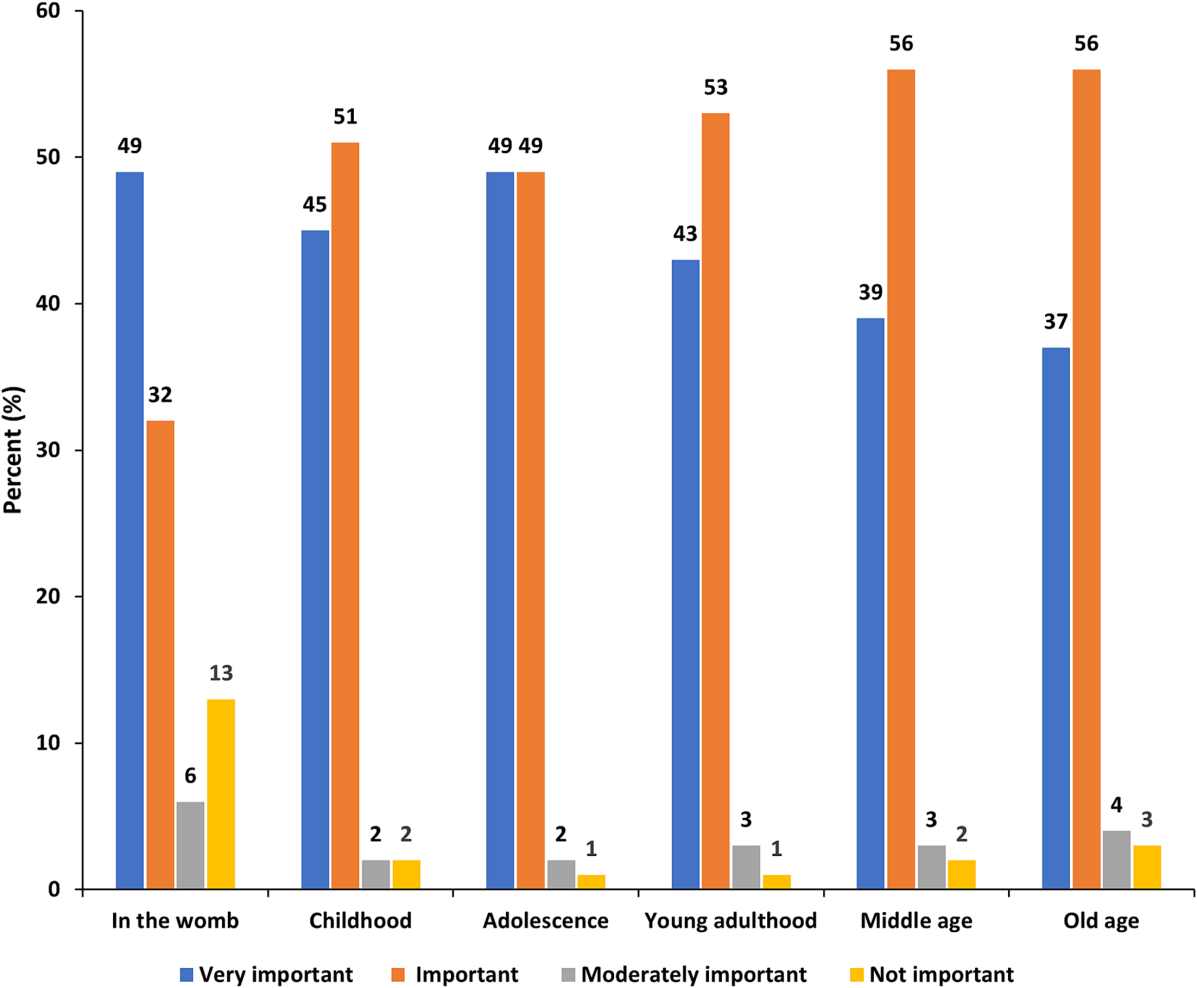
Perceived important stages in life to take special care of the brain.

The popular stages in life in which taking special care of the brain is very important as believed by the participants were in the womb (49%) and during adolescence (49%), followed by during childhood (45%) (Figure 4). The least mentioned life stage considered very important for special care of the brain was during old age (37%).

Older participants (above 40 years), as compared to the younger ones, attached more importance to taking special care of the brain in the womb (OR = 3.06, 95% CI = 1.82–5.43), during young adulthood (OR = 2.59, 95% CI = 1.01–8.02), during middle age (OR = 2.95, 95% CI = 1.10–10.3), and old age (OR = 3.93, 95% CI = 1.73–10.6) (Table 5). Females, as compared to males, give more importance to adolescence (OR = 3.96, 95% CI = 1.30–13.4) and young adulthood (OR = 2.19, 95% CI = 1.02–4.67) for special care of one’s brain. Having healthcare experience was significantly associated with the opinion that is important to take special care of the brain right in the womb (OR = 1.56, 95% CI = 1.07–2.30).

**Table 5:** Association between demographics and perceived important stages in life to take special care of the brain.

Factors	In the womb		Childhood		Adolescence	
	OR	95% CI	OR	95% CI	OR	95% CI
Age						
18–40	1.00		1.00		1.00	
>40	**3.06**	**1.82, 5.43**	1.19	0.45, 3.53	3.23	0.81, 21.6
Gender						
Men	1.00		1.00		1.00	
Women	1.12	0.74, 1.68	1.90	0.76, 4.71	**3.96**	**1.30, 13.4**
Education						
Lower	1.00		1.00		1.00	
Higher	1.10	0.76, 1.59	1.54	0.64, 3.68	2.55	0.84, 8.54
Healthcare experience						
No	1.00		1.00		1.00	
Yes	**1.56**	**1.07, 2.30**	1.03	0.44, 2.52	0.38	0.11, 1.11

Factors	Young adulthood		Middle age		Old age	
	OR	95% CI	OR	95% CI	OR	95% CI
Age						
18–40	1.00		1.00		1.00	
>40	**2.59**	**1.01, 8.02**	**2.95**	**1.10, 10.3**	**3.93**	**1.73, 10.6**
Gender						
Men	1.00		1.00		1.00	
Women	**2.19**	**1.02, 4.67**	0.92	0.40, 1.94	1.63	0.90, 2.89
Education						
Lower	1.00		1.00		1.00	
Higher	1.18	0.56, 2.46	0.97	0.47, 1.91	1.04	0.59, 1.80
Healthcare experience						
No	1.00		1.00		1.00	
Yes	0.85	0.41, 1.79	1.22	0.62, 2.52	1.31	0.75, 2.35

OR, odds ratios; CI, confidence intervals.

OR are adjusted for age, gender, education and healthcare experience.

Bold values are significant at *P* < 0.05.

## Discussion

This is the first brain health survey conducted in the UAE aimed to investigate and understand the perception of people around brain health. This study was designed based on the Global Brain Health Survey developed originally by Lifebrain Consortium and conducted in Europe with over 27,000 participants.

The findings of the study showed that there is a general awareness in the UAE around the importance of sleep, lifestyle choices, and avoiding substance abuse for brain health. However, there are variations based on age, gender, and education. Older individuals tend to prioritize different activities and factors for brain health compared to younger ones. Additionally, gender differences highlight varying perceptions and practices. Both males and females prioritized having enough sleep for brain health, balancing professional and family life, performing religious activities. With women less likely to engage in certain activities such as taking nutritional supplements and practicing relaxing activities. Older participants showed more tendency to follow healthier lifestyle to preserve their brain health and to follow safety recommendations such as wearing helmets, and believe in the influence of sleeping habits, physical environment, family history, and family income on brain health. Also, the study revealed that women are more likely to believe in the influence of social environment, while having higher education is associated with the perception of various factors strongly influencing brain health. The study provides valuable insights into the factors considered significant for maintaining and enhancing brain health in a diverse population.

The findings of our study are in line with those of the original study carried out in 2020, with substance use ranking high on the factors that influence brain health, and are consistent with the result of GBHS conducted in Australia and the US (9, 17, 18). In present study, the most influential factor on brain health, reported by a high percentage of both male and female participants, was substance use, followed by sleep habits, social environment, physical health, and religious activities, in that order. Physical activity was seen as one of the highly influencing factors among the respondents which was at par with the findings in the GBHS survey (9). Our participants regarded sleep as a highly important factor for brain health, which was in line with previous studies conducted in the UK on the importance of sleep and brain health (19).

Females regarded the influence of social environment over brain health, higher than males. An interesting finding of the paper was the influence of family income on the brain health between men and women, where it was noted that women associated higher family income with lower chances of negative impact on brain health. We observed a difference in responses between male and female participants on the account of monthly income as a factor that influences brain health, with men regarding monthly income much higher than women (which was the least perceived factor for women). A study conducted in the US by Gresenz et al. showed a strong association between mental health and income levels, and the correlation of individual income and mental health (20). Another study assessing the association of mental health and income found no association between income and mental health disorders in Latin countries (21).

Factors that were also viewed to influence brain health were diet, education, profession, physical activities and religious activities in decreasing order, amongst others. Our results showed that genetics accounted for the second least perceived factor to influence brain health among the most common factors, where studies conducted by Friedman et al. have shown genetics to be the most identified risk factor for dementia and Alzheimer’s disease (22). The responses to the study questions about diseases or conditions believed to be associated with brain health showed that the respondents were aware of schizophrenia, epilepsy, or depression to be associated with brain health, but had low awareness about the association of high cholesterol and thyroid disease with brain health.

The perceived important stages in life for the brain were seen to be in the womb or fetal stage, which was similar to the finding of the GBHS survey- followed by adolescence and childhood (9). Adolescence was believed to be viewed more important stage for taking special care of brain by men than women. Participants who were younger than 40 years were more likely to perceive brain development in the womb as an important stage for taking care of the brain. It is still unclear the participants’ understanding of “in the womb” as fetal development of brain or as a period of life for taking care of brain health for the mother’s brain.

Religious activities were viewed to be the most frequently done activity to improve brain health, followed by physical exercise and socializing. Earlier studies have shown a relationship between religious and spiritual involvement (23, 24), physical activities (25, 26) and improved cognitive functioning and overall health benefits. Participants with higher education viewed certain factors to influence brain health more like social environment, physical health and having goals in life, compared to those with low education. Previous studies have reported associations between education and cognitive improvement with education influencing decisions and lifestyle (24). Men and women had different outlooks on quite a few activities that are done to improve brain health which is depicted by different interests and different life styles. Gender differences were always a focus point in studies around the world when it comes to disease development, lifestyle and health behaviors as well as seeking help and prevention (27). Previous studies have shown that women may fear cognitive decline and Alzheimer disease more than men (27), however, in our study we did not investigate people’s fear and apprehension rather that than their action and practices as they report it.

The way to reduce health disparities and increase awareness about brain health from a community point of view is by familiarizing the knowledge about dementia, mental health and cognitive function to the public before brain and neurological diseases manifest in an aging population (18). Drissi et al. (28) conducted a study to understand the attitudes around using information and communication technology to deliver connected mental health solutions to the service users by building a framework centered around maintaining anonymity, offering accessible mental health and psychiatric evaluation services, being available in different languages and being culturally considerate. Furthermore, the importance of cultural factors in the perception and attitude of service users toward mental health and brain health are also reported (28, 29). A study conducted in Middle east, North Africa and south Asia region noted explicitly the knowledge gap in aspects of limited epidemiological data, limited resources and limited fieldwork in research and awareness around Parkinson’s disease, taking into account the region and its environmental factors such as rates of obesity and cardiovascular diseases, role of genetics, low awareness about brain health and brain disorders among the population (30).

The results of a study to assess the attitudes of Qatari students toward treatment of mental illnesses found that over 80% of respondents did not want to seek professional help to treat their mental illness and would prefer speaking to family and friends about it to gain support (31). Similarly, the findings of a study to understand public perceptions of Emirati population toward epilepsy showed that the majority of the population did not have good understanding about epilepsy and seizures compared to western countries (32).

Over the years, the UAE has significantly improved accessibility to mental health services and has been strategically expanding them across the country (33). However, there remains a substantial need to raise awareness and reduce the stigma associated with mental health conditions. The results of our study highlight the need of culturally tailored brain health initiatives which may include public awareness campaigns and interventions addressing specific demographic groups. Additionally, it is crucial to integrate brain health education in the healthcare systems, and screening of brain health risk factors particularly for patients with chronic diseases or those at risk of cognitive decline. As part of the preventive strategy, policy makers need to address brain health as a priority agenda. Future research including longitudinal studies to track the changes in brain health perceptions across diverse groups, and comparing the results across regions may better inform national and global policies to mitigate the impact of brain health diseases and improve public health outcomes.

### Strengths and limitations

Our study strengths include being conducted on the general population of UAE, which constitutes Emirati nationals and expatriates living in the UAE. Moreover, our sample size was large and has covered responses from both Emiratis and expats. The study was translated in both Arabic and English, hence it was accessible to people who spoke either of these two languages, keeping in mind that the Emiratis speak Arabic, while 85% of expats use English as a form of communication (34). Our study covered both cognitive and mental aspects of brain health, as it was a continuation of the pilot GBHS conducted by Lifebrain in 2019–20. The present study questionnaire was developed using a similar questionnaire that was used in the GBHS while maintaining the rigor of the survey questions and objectives. By adopting the GBHS adding additional questions, we demonstrate the cross-cultural applicability and contribute to global understanding of how cultural, social, and demographic factors may influence brain health attitudes.

One of our study limitations was that 42% of the study participants worked in healthcare, hence the results may not be representative of the general population of the UAE with regards to brain health, limiting the generalizability of our results. Another limitation is using a convenience sampling strategy, which can introduce selection bias. We adopted the convenience sampling due to practical constraints of reaching a geographically dispersed population within the UAE, which allowed us to collect the data efficiently from a diverse pool of participants. Additionally, we made efforts to ensure broad outreach by targeting multiple platforms and locations. Another limitation was the lack of consideration of people who could not access an online survey or were not as digitally connected since the survey relied on social media and scanning of QR code. However, in spite of the limitations, the survey was able to reach wider audience and the response rate, for a study that was conducted for the first time among the residents in the UAE, turned out to be good.

## Conclusion

Our findings provide valuable insights into how cultural, social, and demographic factors influence the practices and beliefs toward brain health in the UAE, and may contribute to the global discourse on brain health. These results advocate the need for culturally sensitive interventions, particularly for younger individuals, women, and those with varying educational backgrounds. These insights may inform public health strategies in the UAE and similar multicultural, rapidly aging societies, and may help to enhance global brain health research.

## Data Availability

The raw data supporting the conclusions of this article will be made available by the authors, without undue reservation.
