# Supplementary material for: Perceptions about brain health among the United Arab Emirates population using the global brain survey: a cross-sectional study
Source: Front Public Health. 2025 Mar 11;13:1518938. doi: 10.3389/fpubh.2025.1518938 (PMC11932854; doi:10.3389/fpubh.2025.1518938)
Supplement: Supplementary file 1 [file Supplementary_file_1.docx]

**Supplemental online material**

**Supplemental Table 1.** Activities done purposefully for brain health.

**Supplemental Table 2.** Factors perceived to influence brain health strongly

| **Supplementary Table 1.** Activities done purposefully for brain health | | | | |
| --- | --- | --- | --- | --- |
| **Activities** | **Frequently** | **Occasionally** | **Rarely** | **Never** |
| Eat healthy | 448 (36%) | 528 (44%) | 166 (13%) | 91 (7%) |
| Exercise | 333 (27%) | 540 (44%) | 258 (21%) | 102 (8%) |
| Sleep enough | 603 (49%) | 475 (39%) | 114 (9%) | 41 (3%) |
| Relaxing activities | 300 (24%) | 529 (43%) | 261 (21%) | 143 (12%) |
| Profession-Family life balance | 348 (28%) | 655 (53%) | 170 (14%) | 60 (5%) |
| Wear helmet | 228 (18%) | 230 (19%) | 190 (15%) | 585 (48%) |
| Nutritional supplements | 241 (20%) | 515 (42%) | 228 (18%) | 249 (20%) |
| Socialize | 453 (37%) | 552 (45%) | 160 (13%) | 68 (5%) |
| Religious activities | 617 (50%) | 403 (33%) | 127 (10%) | 86 (7%) |

| **Supplementary Table 2.** Factors perceived to influence brain health strongly | | | | | |
| --- | --- | --- | --- | --- | --- |
| **Factors** | **Very strong  influence** | **Strong influence** | **Moderate  influence** | **Weak  influence** | **No  influence** |
| Physical health | 600 (49%) | 481 (39%) | 124 (10%) | 19 (1.5%) | 8 (0.5%) |
| Diet | 466 (38%) | 521 (43%) | 191 (16%) | 30 (2.5%) | 7 (0.5%) |
| Physical environment | 453 (37%) | 481 (39%) | 239 (19%) | 44 (4%) | 9 (1%) |
| Social environment | 669 (54%) | 404 (33%) | 135 (11%) | 16 (1.5%) | 4 (0.5%) |
| Education | 523 (42%) | 494 (40%) | 192 (16%) | 17 (1.5%) | 6 (0.5%) |
| Profession | 444 (36%) | 528 (43%) | 222 (18%) | 33 (2.7%) | 5 (0.3%) |
| Family income | 437 (35%) | 341 (28%) | 341 (28%) | 87 (7%) | 27 (2%) |
| Genetics | 403 (33%) | 448 (36%) | 292 (24%) | 70 (6%) | 18 (1%) |
| Family medical history | 383 (31%) | 466 (38%) | 283 (23%) | 67 (6%) | 28 (2%) |
| Substance use | 926 (75%) | 228 (19%) | 46 (3.7%) | 4 (0.3%) | 28 (2%) |
| Sleeping habits | 712 (58%) | 390 (32%) | 106 (7%) | 17 (2%) | 7 (1%) |
| Having goals | 465 (38%) | 496 (40%) | 230 (19%) | 30 (2%) | 10 (1%) |
| Religious activities | 488 (40%) | 448 (36%) | 232 (19%) | 40 (3%) | 25 (2%) |
